# How The Challenge Initiative Adapted and Used Pause and Reflect Responsive Feedback Sessions for Adaptive Management in Nigeria

**DOI:** 10.9745/GHSP-D-22-00209

**Published:** 2023-12-18

**Authors:** Nneoma Anieto, Lekan Ajijola, Victor Igharo, Sarah Jane Holcombe, Lisa Mwaikambo

**Affiliations:** a The Challenge Initiative, Nigeria Hub, Johns Hopkins Center for Communication Programs, Abuja, Nigeria.; b The Challenge Initiative, Bill & Melinda Gates Institute for Population and Reproductive Health, Baltimore, MD, USA.; c The Challenge Initiative, Johns Hopkins Center for Communication Programs, Baltimore, MD, USA.

## Abstract

**Background:**

The Challenge Initiative (TCI) supports state governments to effectively and sustainably scale up family planning and reproductive health (FP/RH) programming in Nigeria. Given the limited evidence on successful scale-up of health interventions, TCI has established responsive feedback (RF) approaches to regularly review and reflect upon its strategies to quickly adapt and document lessons for scaling FP/RH interventions. One of the RF components adopted was pause and reflect (P&R) exercises to facilitate adaptive management.

**Methods:**

TCI conducted quarterly P&R exercises to identify what works, adapt strategies where needed, and document lessons learned. These exercises were typically conducted as focus group discussions where staff members deliberated on a topic, strategy, or action and discussed how best to refine, diffuse, or discard it. About 10–15 staff participated in each session, including country office technical advisors, state program managers, and technical leads.

**Results:**

TCI has conducted 4 P&R exercises to date. The first P&R focused on identifying effective strategies for scaling up FP/RH interventions and led to the recognition of TCI's coaching strategy and FP in-reaches as evidence-based approaches. The second P&R focused on how to improve TCI's Reflection and Action to Improve Self-reliance and Effectiveness tool to effectively measure governments' capacity to implement FP/RH interventions. The third P&R on graduation activities of TCI's first phase states revealed best practices for planning graduation activities for its second phase states. The fourth P&R on TCI's coaching strategy showed that geographies require a more structured coaching plan to effectively manage their coaching interventions. Implementation of identified actions from the P&R exercises contributed to noticeable improvements in programming.

**Conclusion:**

The P&R exercise has contributed to improvements in adaptive management in TCI programming in Nigeria and is recommended for use by implementing partners, government officials, and other community stakeholders as a useful RF tool.

## Abstract (precis)

Through pause and reflect sessions, The Challenge Initiative has instituted improvements for scaling up evidence-based, government-led FP/RH interventions, including government self-reliance assessment and development of more systematic coaching plans.

## Teaser

**Key Findings:**

- Institutionalization of pause and reflect (P&R) sessions as a responsive feedback (RF) mechanism within The Challenge Initiative project in Nigeria has contributed to improved program strategies and applicable lessons learned for replication by others scaling up evidence-based FP/RH interventions.
- Initial challenges to institutionalizing P&R sessions can be addressed with advanced planning, use of an experienced facilitator, and application of RF reflections for adaptive management.

**Key Implications:**

- The P&R exercise is a useful component of RF for adaptive management for implementing partners, government staff, and other program managers and implementers, especially in the context of scaling up health and development interventions through government structures.
- The P&R exercise, unlike monitoring and evaluation techniques, is relevant for uncovering lessons, failures, and areas for improvement, which, when addressed in a timely manner, can contribute to improved programming.

## BACKGROUND

As local governments and partner organizations supporting these governments scale up evidence-based health interventions, it is imperative that they “manage adaptively through continuous learning.”^1^ Responsive feedback (RF) approaches call for “timely assessments that provide actionable feedback to implementers to course correct and achieve intended outcomes” and build in regular interactions between diverse stakeholders, including but not limited to project designers, implementers, researchers, decision-makers, and clients.^2^ Pause and reflect (P&R) sessions can be a key mechanism to facilitate program development and strengthening based on evidence.^2^

RF is especially important in the context of programs aiming to sustainably scale interventions with impact, such as The Challenge Initiative (TCI). Relying solely on outcome measures (e.g., health management information systems data) that either arrive long after management decisions have been made or that do not provide sufficiently detailed insights into how well systems are functioning means programs will miss critical and time-sensitive information needed for course corrections. Ensuring intentional reflection points during the program life cycle and collecting insights from multiple stakeholder perspectives are key inputs in enabling learning that helps guide action toward goals.^3^^–^^6^ Reflection also enables teams to explore their and others' thinking as they experiment with solutions, adjust actions, and reframe problems.

Drawing on Kolb^7^ and Schön,^8^ many argue that reflection is important for effective learning and performance. Faller explains this connection further^9^:

*Reflection is linked to action—past actions that shape meaning-making; present action that embodies knowing; or imagined future action that influences direction and intention.*

In a complex, constantly changing environment, reflection is needed for deep learning, problem-solving, and innovation. For example, if we react quickly, we act on limited information, biases or beliefs, or generic popular practices. Reflection enables us to engage in sense-making and sense-giving to share and compare interpretations in combination with action.

TCI is a platform that supports local governments in urban areas to design, implement, monitor, and rapidly scale up evidence-based family planning and reproductive health (FP/RH) interventions that were proven to work in urban settings in India, Kenya, Nigeria, and Senegal.^10^ The evidence supporting these interventions was generated from the earlier implemented Urban Reproductive Health Initiative, which was rigorously evaluated to show impact among the urban poor cities and over time.^11^ Building upon the demonstrated impact of the Urban Reproductive Health Initiative, the purpose of TCI is to demonstrate feasibility, impact, and sustainability of the interventions as they are scaled up by local governments. TCI regional hubs located in East Africa, Francophone West Africa, India, and Nigeria—and recently expanded into the Philippines and Pakistan—coach local governments as the implementers of the interventions, unlike traditional development programs and the Urban Reproductive Health Initiative, where international nongovernment organizations led the implementation.^12^

It is critical to have tools and processes to iteratively learn, adapt, and change as needed when scaling interventions.^12^^–^^14^ Therefore, TCI uses RF as an essential tool for adaptive management to monitor implementation, institutionalization, continuous improvement, and scale-up of government-led FP/RH interventions. Specifically, TCI has adopted a “learning by doing” approach that includes regularly scheduled P&R sessions in concert with other adaptive management approaches to monitor and provide insights into the programmatic context of program impacts across 111 cities in 11 countries. This includes the ongoing collection of Most Significant Change stories, a complexity-aware monitoring and evaluation technique that compliments performance monitoring by tracking the unpredictable^1^ (described in detail in Ohkubo et al.^5^) and the implementation of the Reflection and Action to Improve Self-reliance and Effectiveness (RAISE) self-assessment tool (described in detail in Malik et al.^14^ and Ajijola et al.^15^). Most public health programs in Nigeria do not use RF and/or adaptive management approaches to iteratively improve programs throughout their life cycle.

In this article, TCI documented the best practices of its application of quarterly P&R sessions as a key element of an RF approach. The adoption of P&R was critical in interrogating key components of TCI's theory of change, which assumes that ownership, financial incentives, and coaching will enable government counterparts to effectively implement and scale up evidence-based FP/RH interventions. P&R sessions provided TCI staff a regular opportunity to prioritize areas where the program would benefit from learning. This enabled TCI to continue to improve its financial and coaching support to governments to ensure effective scale-up and diffusion of the evidence-based FP/RH interventions.

## HOW TCI OPERATIONALIZES P&R SESSIONS IN NIGERIA

The goal of TCI's P&R sessions is to improve how we do our work by identifying promising practices, learning from mistakes, and avoiding future pitfalls in providing coaching support to local governments in implementing evidence-based FP/RH interventions. TCI's quarterly P&R sessions have been largely internally focused and conducted by TCI's regional hubs, TCI's global headquarters, and some local governments (or in the case of Nigeria, state governments). TCI uses a variety of learning techniques to reflect upon what is working well and what can be improved in operationalizing TCI's model and to quickly alter implementation strategies accordingly.

TCI's P&R sessions aim to improve how we do our work by identifying promising practices, learning from mistakes, and avoiding future pitfalls in providing coaching support to local governments in implementing evidence-based FP/RH interventions.

TCI global provided the regional hubs with broad guidance on how to conduct a P&R session using a menu of exercises to select from (Box). Each regional hub selects which approach to use at each quarterly P&R to best suit its needs, given its specific context, and what it hopes to learn so that it can improve its support to local governments to sustainably scale up evidence-based FP/RH interventions. In this article, we share TCI Nigeria's experience and lessons learned from employing P&R sessions to facilitate RF and adaptive management.

Each regional hub selects which approach to use at each quarterly P&R to best suit its needs and what it hopes to learn so that it can improve its support to local governments to sustainably scale up evidence-based FP/RH interventions.

BOX Pause and Reflect Session Possible Exercises

- **Focus group discussion:** A facilitated group discussion among The Challenge Initiative (TCI) staff intended to identify positive and negative changes due to TCI programming that will help to inform adjustments in programming strategies and decisions. This guidance draws upon the most significant change technique and questions that the hubs use in interviewing key local government and community stakeholders (such as what worked well and why and what did not work well and why).
- **After-action review:** A structured review process or debrief—usually a meeting—for project teams to reflect on an event or task they have just accomplished and analyze what happened and why, what worked well, and what can be done better or differently in the future.
- **Data review:** In “coming up with headlines,” participants are asked what they would write for the front page of a newspaper based on their key takeaways from project data that they are reviewing, which may include both positive and negative findings.
- **Fail fair/failure panel:** Stigma-free recounts of events that individuals consider to be failures. Colleagues can draw lessons from sharing these “failure stories” and apply the lessons to their work on similar projects. Unlike either a formal or informal after-action review, information shared during a fail fair is presented in a very relaxed, fun environment.
- **Learning exchange visits/study tours:** A visit or series of visits to 1 or more countries or sites by an individual or group with a specific learning goal in mind. Participants learn firsthand from the experience of their peers how a challenge was solved or a solution was implemented.
- **Peer assist:** A facilitated in-person or virtual event in which peers with relevant experience share their knowledge and experience, usually in the form of best practices and lessons learned, with a team that has requested help on a specific problem, project, or activity.

TCI Nigeria's knowledge management officer (KMO) leads the quarterly P&R sessions. TCI staff based in the 13 states supported by TCI, along with technical leads for advocacy, demand generation, service delivery, monitoring and evaluation, and other program and operations staff based in Abuja, participate in the sessions. State government team members are not typically part of all P&R sessions due to its TCI regional hub-led approach. However, recommendations and corrective actions from the exercise are discussed with them, and the TCI team, led by the state program coordinator, provides them with support to lead relevant corrective actions. Between 10 to 15 people participate in the quarterly sessions, with each session lasting about 90 minutes.

TCI has plans to eventually cascade the P&R sessions to the state level where the state TCI team and their government counterparts will convene and reflect on identified topics quarterly. Some of the benefits of involving government teams in leading the P&R sessions include governments taking ownership of the approach for improving their programs, having a better understanding of the evidence-based FP/RH interventions and the unique contextual factors that impact their effectiveness, undertaking smoother program shifts based on the outcome of the exercise, and planning eventual scale-up of the TCI model to include other primary health care programs beyond FP. Two main constraints to having a fully government-led P&R session include finding the time amid busy schedules and fostering an open and respectful culture that promotes learning and course correction over assigning blame. Government teams usually find it difficult to make the time to openly identify, discuss, and agree that possible interventions and approaches are not working or have failed and discuss the underlying issues that contributed to this in a nonjudgmental way. As a result, government teams often continue to implement interventions and approaches without critical analysis as to what is and is not working and why. To address these constraints, TCI will identify “learning” advocates among government teams and train them to understand that P&R sessions are intended to improve their programs, ultimately achieve desired results, and facilitate best practices.

First, the KMO sends a call for topics relevant to TCI's program implementation to be discussed at upcoming P&R sessions. The topic selected needs to meet the following 2 criteria: high relevance across the project (so that all staff can learn from the discussion) and timely (something that has recently taken place or been observed recently and has ongoing implications for activities). After the topic for reflection is selected, the KMO provides the team members guidance about the session ahead of time (e.g., discussion questions for the focus group discussion [FGD] or after-action review [AAR]) to ensure deeper reflection by participants during the P&R. The KMO facilitates the discussion, records the team's responses, synthesizes the notes, and presents recommendations from the session in a report, which is circulated within 2–3 weeks to all TCI staff who are responsible for incorporating the recommendations into work plans and implementing them with the local government. The KMO has been trained on the use of P&R for adaptive management and best practices for facilitation of P&R sessions. As a result, the KMO understands the importance and possesses the skills to ensure the active participation of all team members and elicit responses, whether positive or negative, from team members. In the absence of a KMO, a trained facilitator can elicit relevant responses during a P&R session. Clients are not part of the P&R exercises, but their concerns are shared by TCI staff members who not only work closely with but also sit with government teams within the state ministries of health and primary health care development agencies and provide support for program implementation. These team members work together with state and local government teams to interact with clients and share challenges noted by clients with respect to service provision. This feedback is often collected as part of the ongoing Most Significant Change story collection technique, anecdotally during community mobilization, neighborhood campaigns, community outreaches, and facility-based in-reaches, and through periodic surveys, such as client exit interviews, which allows the TCI and government teams to analyze and discuss feedback to improve programming.

TCI uses different exercises to conduct the P&R session based on the theory of change area, strategy, or action being reviewed. FGD was the first strategy used because it was necessary to investigate which areas and strategies required reflection and improvement. After identifying a range of strategies that needed reflection for potential improvement, another FGD was conducted on an identified topic during the second P&R session.

The AAR method was used once after a major event where the first set of supported state governments graduated from TCI's direct support after achieving self-reliance. The AAR method is the most appropriate for assessing performance after an activity so that the team can identify what worked well and what could be improved before conducting the activity again. The AAR has been particularly useful for activities that are repeated, like TCI's graduation activities and the implementation of evidence-based interventions, such as FP in-reaches and 72-hour clinic makeovers.

When TCI Nigeria first started to use P&R sessions for RF in 2019, it faced several challenges, including inconsistent frequency of the sessions and a lack of appreciation of their value. As a result, in 2020, TCI Nigeria devised and implemented innovative corrective actions to tackle key challenges identified to ensure that P&R sessions contribute to program improvements (Table 1).

**TABLE 1.** Challenges and Corrective Actions to Ensure Use and Effectiveness of P&R Sessions in Nigeria

Challenges in Implementing P&R Sessions	Corrective Actions Implemented
TCI staff have competing priorities leading to their inability to dedicate time and participate in the P&R session	Made P&R sessions part of end-of-quarter reporting processes and made attendance mandatory. The P&R sessions are now on everyone's calendar well in advance of the session itself and as a reoccurring meeting, so that team members can plan accordingly.
Staff travel and geographic dispersion affected staff availability for the P&R sessions	Transitioned to virtual P&R sessions to allow relevant participants to participate effectively, irrespective of their current locations. This became essential during the COVID-19 pandemic.
Occasional difficulty in selecting reflection areas due to dissenting opinions	Shared with staff the criteria for selection of topics (i.e., cross-project relevance and timeliness), examples of currently emerging themes, new strategies, and challenges reported or observed. Developed a schedule of reflection topics to guide implementation.
Initial reluctance among staff to discuss challenges or failures due to perceived fears of management sanctions	TCI management oriented the team on the importance of discussing failures to ultimately achieve success. Used an experienced facilitator, usually the TCI KMO, to address perceived participant concerns, ensure active participation, and elicit open dialogue.

Abbreviations: KMO, knowledge management officer; P&R, pause and reflect; TCI, The Challenge Initiative.

To date, TCI Nigeria has held 4 P&R sessions since implementing the corrective action strategies to mitigate challenges. These include using 3 FGDs (2 virtual and 1 in-person) and 1 AAR as part of quarterly P&R sessions.

## Content and Results of TCI Nigeria P&R Sessions

### Identifying Effective Strategies for Scale-Up and Systems Strengthening

The first P&R session, conducted in the fourth quarter of 2020, used the FGD approach and set the tone for subsequent P&R sessions. During the session, the team examined its operations and identified its most beneficial strategies for supporting states to scale up FP/RH interventions. The team also identified strategies that needed to be improved or modified, as well as lessons learned during program implementation at the headquarters/Abuja level and in TCI-supported states. Finally, the P&R session uncovered the need for a deeper dive into the design and use of TCI's RAISE tool, which was scheduled for the next P&R session. Actions taken as a result of this P&R exercise have contributed to the scale-up of evidence-based interventions across supported states (Table 2).^16^^,^^17^ For instance, more states are now implementing in-reaches leading to increased uptake of FP services. In-reaches entail the provision of FP services in a designated health facility on selected days after mobilization of clients from the community. The approach contributes to increasing access by removing barriers to uptake of FP, including non-completion of referrals, unavailability of FP methods, absence of trained providers, and lack of consumables leading to charges for services.

**TABLE 2.** P&R Session to Identify Effective Strategies for Scale-Up and Systems Strengthening

Key Insights	Actions Taken
The TCI lead, assist, and observe coaching approach that allows government teams to learn during TCI lead phase, partially lead during TCI assist phase, and fully lead during TCI observe phase is effective in building local government's capacity for self-reliance in FP programming.^16^	Continued scale-up of the TCI lead, assist, and observe coaching model within current states, expanding into more local government areas.
FP in-reaches were identified as an effective strategy for increasing access to FP services among women, including adolescents and youth, as evidenced by increasing service usage data on the health management information system.	Implemented learning exchange visits where states performing optimally as a result of in-reaches provided step-by-step guidance on the implementation of in-reaches to other states to enable them to implement the intervention.
RAISE assessments effectively assess the state government's capacity to implement FP/RH programs, but there is a need to review the tool to make it more user-friendly and less cumbersome.	Scheduled the next P&R session on the RAISE tool and assessments to enable the team to have a deeper discussion on the tool and identify areas for improvement.
Use of advocacy core groups catalyze state governments to release domestic funds for FP program implementation; however, TCI-supported states have achieved different levels of financial release based on the capacity of their respective advocacy core group.^17^	Strengthened the capacity and functionality of the advocacy core groups with closer tracking of financial releases at the state level to monitor impact.

Abbreviations: FP, family planning; P&R, pause and reflect; RAISE; Reflection and Action to Improve Self-reliance and Effectiveness; RH, reproductive health; TCI, The Challenge Initiative.

During the first P&R session, the team examined its operations and identified its most beneficial strategies for supporting states to scale up FP/RH interventions.

Actions taken as a result of the first P&R exercise have contributed to the scale-up of evidence-based interventions across supported states.

### Reflecting Upon TCI's RAISE Tool to Ensure State Ownership and Sustained Capacity

The second P&R session, conducted in the first quarter of 2021, examined the design and use of the RAISE tool, a management and organizational tool that uses an RF approach to help local governments to assess their readiness to sustain implementation of FP/RH interventions. Using the FGD approach, the team discussed the relevance of the RAISE tool in the TCI-supported states, the impact of the RAISE assessments on improving FP/RH interventions in the states and strengthening the states' coordination and implementation of FP/RH activities, and the operationalization of RAISE in terms of the frequency of conducting the assessments and relevant steps to take to ensure that states use the results of the assessments for adaptive management. Implementation of actions from this P&R session led to modifications to the RAISE tool and wider visibility for the tool, for example, the identification of state government leads to track implementation of the action plan (Table 3) and recognition of TCI Nigeria on its outstanding use of the RAISE tool for RF.

**TABLE 3.** P&R Session on RAISE Assessments

Key Insights	Actions Taken
States need greater support to disseminate RAISE findings to ensure relevant stakeholders are updated on progress and supportive of the action plans derived from the assessment findings. Besides email, RAISE findings could also be disseminated through technical working group meetings, contraceptive technology update meetings, and other relevant stakeholder meetings.	Supported states to conduct formal dissemination of findings from RAISE assessments through joint development of slides and printing of dissemination materials. Identified government leads to implement recommendations from RAISE assessments at the state level within stipulated timelines.
The action plan component of the RAISE tool should be modified to allow state teams to clearly articulate their technical assistance needs and to identify a state government lead who would be responsible for tracking implementation of action plan activities by responsible persons.	Updated the RAISE tool action plan component and simplified it for ease of reflection on technical assistance required alongside responsible persons and implementation timelines.
Innovative ways to triangulate RAISE assessment results with client volume trends for the period under review should be explored.	Triangulated data from RAISE assessments with other data sources like a health management information system and project data to further inform program improvements. Kept the RAISE dashboard at the state level to enable TCI and government teams jointly evaluate the use of the tool and monitor scores closely.
Guidance should be provided to states to select the right mix of stakeholders to participate in the RAISE assessment, conduct orientation on the application of the tool and clarify expectations addressing key issues raised above. As much as possible, the same set of participants should be maintained, and the small consensus groups should be evenly distributed based on the domain areas to support an objective assessment.	RAISE facilitators engaged relevant state coordinators to follow the guidance on the RAISE tool for participant selection to ensure the right mix and representation of teams for objectivity in review and scoring.
RAISE assessment should be conducted quarterly for the first 2 quarters and biannually subsequently to give adequate time for identified issues to be resolved before a new assessment.	Implemented RAISE assessments quarterly for states receiving active support from TCI and biannually for states that have graduated from the TCI program.

Abbreviations: P&R, pause and reflect; RAISE; Reflection and Action to Improve Self-reliance and Effectiveness.

### Reflecting on Activities to Mark Graduation of States From Direct TCI Support

The third P&R exercise, conducted in the third quarter of 2021, was an AAR of the team's activities to mark the graduation of its first-phase states from receiving direct TCI technical and financial support. Five Nigerian states graduated from TCI's direct support after receiving 4 years of technical and financial assistance to implement evidence-based FP/RH interventions. The team secured an external moderator to facilitate this P&R session to ensure every team member had the opportunity to contribute to the discussion, given that the KMO was heavily involved in implementing the activities under reflection. After the P&R session, a list of documented recommendations was shared with the second-phase states to guide their graduation activities, which occurred in the second quarter of 2022. For example, the team recommended planning for the graduation activities at least 1–2 months before the graduation event itself and developing an itemized timeline and schedule to ensure timely deliverables (Table 4).

**TABLE 4.** Recommendation Generated at the P&R Session on Graduation Activities in TCI States

**Planning and logistics**	Start planning for graduation events and production of graduation documentation earlier to allow for quality control and more time to review; documents will take at least 1–2 months to finalize. Clearly itemize what needs to be done and come up with a timeline for each task; consider creating a production schedule for each deliverable. Identify needs for additional support (i.e., knowledge management/finance and operations) during the graduation/dissemination event. Work with state government officials as soon as possible to identify individuals who should be recognized/honored during the graduation dissemination events; this is important to ensure no one is overlooked or omitted in the process.
**Budgeting**	Begin planning process early and allow enough time to engage with vendors, including obtaining estimates early enough for appropriate approvals. Work realistically and within the current budget but ensure state budgets include contingency funds for unexpected situations.

### Reflecting on TCI's Coaching Approach

The fourth P&R exercise, conducted as an FGD during TCI Nigeria's annual retreat meeting in the fourth quarter of 2021, focused on TCI's coaching approach. Participants were clustered into 3 FGDs to ensure effective reflection and feedback, considering the larger number of participants that included TCI global staff and additional TCI staff from the states. Each FGD reflected on the same questions: (1) What is working with the current coaching approach? And why/how do we know this? (2) What is not working? And why/how do we know this? (3) How can we make our coaching approach more structured? (4) What parameters should be captured in a new coaching logbook?

Each group had an identified and trained moderator to ensure all participants engaged actively and responded to the questions. Reflections and actions from the groups were collated and shared with the KMO, who harmonized and subsequently disseminated the feedback and final actions to the entire TCI team for adoption and implementation. Key recommendations included having a state-specific coaching plan to guide coaching interventions and ensuring disaggregation of coaching into scheduled and ad hoc coaching types for effective management (Table 5).^18^^–^^23^

**TABLE 5.** P&R Session on TCI's Coaching Approach

Key Insights	Actions Taken
The development of a coaching schedule based on the outcome of the routine FP quarterly supportive supervision and RAISE assessments conducted by the state government team with support from TCI is critical. States that develop the schedule seem to implement coaching seamlessly compared to states that do not. All supported states are recommended to develop quarterly coaching plans and schedules to aid the implementation of coaching.	Supported states that were implementing coaching without a particular structure to develop quarterly coaching plans and schedules to guide their coaching interventions.
Disaggregate coaching into scheduled, on-demand, and ad hoc and ensure reporting to aid TCI management's understanding of state peculiarities and how to help the states to improve knowledge transfer between coaches and coachees.	Disaggregated coaching interventions clearly in reports to identify whether they were scheduled, on-demand, or ad hoc. TCI Nigeria is using this information to further program with the states.^18^
Develop a coaching logbook and ensure that relevant reporting parameters are captured in the logbook.	Developed a coaching logbook that captures relevant indicators, including coach/coachee information, type of coaching, coaching topic, and duration of coaching. This information is relevant for programming.^19^
Institute a more routine means of assessing coachees' satisfaction with the coaching they have received. Currently, TCI implements annual online TCI-U surveys, which measure satisfaction and quality of coaching.^20^	Developed a quarterly coachees' satisfaction checklist that is currently undergoing TCI management's review before roll-out.^21^
Develop more animated videos^22^ on TCI University (TCI-U)^23^ that coaches can use as part of their coaching sessions with stakeholders. The animated videos provide easy steps for the coachees to understand how to replicate a particular approach in their context.	Began efforts to identify and prioritize high-impact FP/RH interventions to convert into animated how-to videos for stakeholders. TCI Nigeria started with the development of an in-reach video.^22^

Abbreviations: FP, family planning; P&R, pause and reflect; RH, reproductive health; TCI, The Challenge Initiative; TCI-U, TCI University.

A key recommendation from the fourth P&R session included having a state-specific coaching plan to guide coaching interventions.

### Monitoring and Evaluation of TCI P&R Sessions

The KMO led collection, collation, analysis, and reporting of P&R exercise outcomes and further synthesized next steps from the reflection and shared them with the broader team for review. After the review by team members and participants, a final report of the P&R exercises with clear actionable items, responsible persons, and timelines was shared with the entire team. The KMO tracked implementation of the action items for the quarter and included progress updates in the monthly report ahead of the next P&R exercise. The KMO has kept a P&R tracker that includes topics reflected on and emerging topics already chosen for future reflection yet to be discussed. This allows team members to refer to pending actions that have yet to be implemented from previous reflections and plan for new reflections. At times, emerging issues have taken precedence over planned reflections, and these have been updated on the tracker. Improvements recorded based on implementation of next steps from P&R sessions were captured in reports and documented in the specific process, strategy, or approach reflected on. In the future, TCI will monitor these improvements from their respective programs and match them to recommended actions on the P&R template as a direct evaluation tool for measuring the impact of P&R exercises. TCI continues to explore improvements in capturing RF mechanisms as a whole, including the P&R exercises.

## LESSONS LEARNED AND RECOMMENDATIONS

### Prioritization by Leadership With Staff Input Required for Buy-in for the P&R Sessions

Instituting P&R sessions for RF in TCI Nigeria came with initial challenges, possibly because of the early focus on “doing the work” over “reflecting and reviewing the work.” Staff members did not initially prioritize the P&R exercise and could not understand its value nor the relevance of the exercise for RF and adaptive management. Making the exercise mandatory for all staff, aligning it with quarterly reporting, and soliciting staff input into the identification of topics for reflection increased staff participation. Conducting P&R sessions virtually also removed the constraints of gathering physically and ensured that staff members based in the supported states could contribute more easily.

The successful implementation of the first P&R session with a report on recommendations and actions taken enabled staff to see the value of reflecting and management's commitment to the recommendations from the P&R exercise. TCI Nigeria staff members now see P&R as an invaluable tool for RF and adaptive management. This was inferred from feedback received from the TCI state team members to the Abuja-based regional team, following the implementation of the recommendations. This realization increased staff willingness to participate in the exercise, share their experiences, learn from their colleagues' experiences, and offer their own recommendations for program improvement.

TCI Nigeria staff members now see P&R as an invaluable tool for RF and adaptive management.

### P&R Sessions Can Lead to More Effective and Efficient Programs

P&R exercises are an effective way to receive feedback from program managers and implementers who are responsible for the day-to-day operations of a program. This feedback often results in targeted implementation of strategies that have been proven to work in supported states or modifications to program interventions, with increased cost efficiency and better outcomes. After understanding how the feedback can improve program activities, staff members overcame their initial reticence and became more willing to scrutinize their efforts and modify according to lessons learned from others on the team more openly.

TCI Nigeria continues to look for ways to improve its P&R sessions by training its state program coordinators to replicate the P&R approach with state government stakeholders. In this case, TCI state program coordinators will serve as the facilitator, like the KMO, and share the report on recommendations and actions taken by the state with TCI headquarters, which will ensure TCI's support is more tailored to fit the needs of each of its supported states.

### Prioritize RF Mechanisms and Create a Conducive Environment to Implement Changes

Given the importance of RF for adaptive management, organizations should prioritize and institute regular RF mechanisms, like quarterly P&R sessions. Participation in P&R exercises should be introduced to staff members as a key requirement for their job; this will ensure that the exercises are given priority and are adequately prepared for by staff. Additionally, virtual solutions can be explored to ensure that participants at P&R sessions are diverse; this will save the cost of inviting participants to sit together in the same physical place.

P&R sessions in themselves do not guarantee change. To capture the process of change, it is important to document and implement the recommendations from the P&R sessions. This should include follow-up actions, feedback, and formal administrative approvals and leadership concurrence for each action proposed and executed. Only then can P&R sessions be successfully incorporated and used for adaptive management.

Given that reflection often requires asking powerful questions and testing taken-for-granted assumptions, having a trained facilitator is critical to creating a safe space, especially given the potential power dynamics in a workplace environment. In addition, it is crucial to have the buy-in of leadership and all staff on the purpose of the P&R exercises, given that reflection can challenge organizational norms and biases. Finally, the P&R sessions must become an integral part of programming with protected time devoted to the approach to ensure its full potential as an RF mechanism. As a result, TCI Nigeria has begun planning for P&R sessions early, identifying potential topics for discussion that align with work plan activities each quarter, and scheduling and setting aside protected time for P&R sessions each quarter. Although TCI Nigeria seeks to better integrate the approach in a systematic way, it also recognizes that the P&R sessions need to be responsive to the specific learning needs of the project at the time to ensure timely adaptive management. New topics may arise or change at the last minute.

## CONCLUSION

In conclusion, TCI Nigeria's P&R sessions have contributed to improvements in program learning and adaptive management in Nigeria, but more needs to be done to address the challenges in making the time and space for such reflection. TCI, as a technical assistance partner working with local governments, has reaped the benefits of RF strategies, such as P&R, and has found them to provide critical feedback as it supports state and local governments to scale access to FP/RH programming. In the future, TCI Nigeria is exploring how to introduce and institutionalize the periodic use of P&R by government managers within state-level coordinating bodies, such as technical working groups, so that they can experiment and adapt it to strengthen their own programming.

## Notes

**Peer Reviewed**

**First published online:** June 7, 2023.
